# Effect of aging on healing of mandibular tooth extraction socket in a rat model

**DOI:** 10.1111/eos.70099

**Published:** 2026-04-30

**Authors:** Kyung Su Shin, Han Wool Kwak, Jong‐Seong Chae, Si‐On Choi, Suah Han, Hwa Young Yu, Dae Ho Leem, Hyun Seok

**Affiliations:** ^1^ Department of Oral and Maxillofacial Surgery School of Dentistry Jeonbuk National University Jeonju Republic of Korea; ^2^ Research Institute of Clinical Medicine of Jeonbuk National University‐Biomedical Research Institute of Jeonbuk National University Hospital Jeonju Republic of Korea; ^3^ Department of Oral Pathology School of Dentistry Jeonbuk National University Jeonju Republic of Korea

**Keywords:** aging, alveolar bone formation, tooth extraction socket

## Abstract

This study evaluated the effect of aging on bone regeneration in mandibular extraction sockets. Male Sprague–Dawley rats were divided into three age groups: 2‐month‐old (2 M, *n* = 6), 10‐month‐old (10 M, *n* = 6), and 18‐month‐old (18 M, *n* = 5). Two weeks post‐extraction, comprehensive analyses were performed using micro‐computed tomography (*µ*‐CT), serum bone turnover markers, histology, and immunohistochemistry. Notably, to ensure accurate volumetric assessment, a specific tooth‐based region of interest utilizing the extracted tooth was employed for precise *µ*‐CT analysis. Radiologically, the 18 M group exhibited significantly reduced bone volume and trabecular thickness compared to the younger groups. Serum analysis revealed that younger rats maintained higher baseline metabolic activity, specifically regarding osteocalcin and CTX‐I, and exhibited a distinct post‐extraction tartrate‐resistant acid phosphatase 5b response. Histological and immunohistochemical findings corroborated these results; the 10 M group demonstrated the most favorable bone regeneration with maximal alkaline phosphatase expression filling the socket, whereas the 18 M group exhibited compromised healing characterized by empty sockets and inflammatory cell accumulation. In conclusion, aging significantly deteriorates the biological healing capacity of alveolar bone. Although younger animals exhibit robust metabolic responses and bone formation, senescent rats show a marked decline in regenerative potential, emphasizing the need for age‐specific considerations in dental rehabilitation.

## INTRODUCTION

Comprehensive bone changes across the entire skeleton owing to aging have been extensively investigated. Changes occur not only in the quantity of bone but also in the quality of the inner face, such as a decrease in the number of osteoblasts and osteocytes with age [1]. Aging has a negative impact on bone metabolism, impairing both healing and regeneration [2, 3]. Studies have investigated the effect of aging on craniofacial bones: Aging delays bone regeneration in the maxillary midpalatal suture in a rat model [4]. In a bilateral condylar fracture rat model, aging is associated with delayed bone healing at the fracture site [5]. In oral and maxillofacial surgery, alveolar bone healing after tooth extraction is important for successful oral rehabilitation [6]. However, the relationship between age and extraction socket healing has been poorly investigated. A comprehensive understanding of this relationship will enable proper oral rehabilitation of older adults.

Tooth extraction is the most commonly performed dental procedure. Following extraction, the fibrinous blood clot and remaining periodontal ligament (PDL) provide a foundation for maturation and healing [7]. During the healing process, blood clots are replaced by granulation tissue, which is subsequently replaced by connective tissue. The remaining PDL fibroblasts migrate into the blood clot, where they form dense connective tissue [8]. These cells then facilitate the formation of new vessels and collagenous fibers. Subsequently, the fibroblasts differentiate into osteoblasts, initiating new bone formation [9]. This bone regeneration process begins 2 wk after extraction and continues for up to 16 wk [8].

Healing of extraction sockets is influenced by various systemic and general disease factors in the host. These are also affected by local factors outside the host. General diseases (such as diabetes) reduce the number of mesenchymal stem cells, impair their differentiation, and induce apoptosis [10]. Diabetes also increases osteoclastogenesis, which results in decreased bone formation [11]. Lack of saliva negatively impacts both soft and hard tissue healing in the extraction socket. It causes a deficiency of nitric oxide, tumor necrosis factor‐α, and prostaglandin E2, delaying healing in the early stages of wound repair [12]. Smoking affects bone remodeling after tooth extraction [13]. Nicotine negatively affects the differentiation and activation of osteoblasts and osteoclast‐like cells, hindering the healing of the extraction socket [14]. Although aging is known to negatively impact extraction socket healing, no study has investigated the precise patterns of bone healing by age.

Bone turnover markers can help evaluate the effect of general condition on the craniofacial bone remodeling capacity [15]. Bone alkaline phosphatase and osteocalcin (OC) are bone formation markers [16]. Receptor activator of nuclear factor (NF)‐kappa B ligand (RANKL) interacts with a receptor of RANK on the surface of the osteoclast precursors. This triggers the fusion of osteoclast lineage, initiates bone resorption, and regulates osteoclast activity by suppressing apoptosis [17]. Serum C‐telopeptide of type I collagen (CTX‐I) is a reference marker of bone resorption [18]. Moreover, osteoclasts secrete tartrate‐resistant acid phosphatase 5b (TRAP5b) into circulation during bone resorption. In this study, we investigated the relationship between the general condition of bone turnover markers and the local state of extraction socket healing in different age groups.

Despite the clinical importance, the precise effects of aging on socket healing following tooth extraction remain unclear. Previous studies on mandibular defects have suggested that the increased basal bone volume (BV) in older animals might mask the decline in biological healing capacity [15]. To overcome this anatomical confounding factor and evaluate the true biological healing potential, we employed a specific “tooth‐based region of interest (ROI)” method that strictly isolates the extraction socket from the surrounding basal bone. Using this precise method, we radiologically and histologically evaluated the amount of regenerated bone in rats of different ages (2, 10, and 18‐month‐old) after mandibular molar tooth extraction. To the best of our knowledge, this is the first study to evaluate extraction socket healing across a serial age range using a tooth‐based ROI approach. Additionally, we evaluated and compared the serum levels of several bone turnover markers in the three age groups.

## MATERIAL AND METHODS

### Animals and materials

The animal experiments were performed under the approvement of the Institutional Animal Care and Use Committee of Jeonbuk National University Hospital, Jeonju, Republic of Korea (JBUH‐IACUC‐2022‐9). Seventeen Sprague–Dawley male rats (Samtako Biokorea, Osan, Republic of Korea) were used in this study. The rats were housed at one rat per cage under standard laboratory conditions. Rats were fed ad libitum and provided a standard rodent diet and water. The experiment was delayed until 2 wk for the adaptation of the animals in the new environment. We divided the rats into the following three groups based on age: first group, 2 months old (2 M) (*n *= 6); second group, 10 months old (10 M) (*n* = 6); and third group, 18 months old (18 M) (*n* = 5).

### Surgical intervention and experimental design

Extraction of the molar tooth was done under general anesthesia. A mixture of tiletamine hydrochloride, zolazepam (Zoletil 15 mg/kg; Vibac), and xylazine (Rompun 0.2 mL/kg; Bayer Korea) was injected intramuscularly. A single surgeon performed all the surgical interventions under aseptic conditions using sterile surgical instruments. Blood samples were collected from the left jugular vein of the rats preoperatively. The surgery involved the extraction of the first or second molar from each rat. Postoperatively, the intramuscular injection of gentamycin (1 mg/kg; Kookje) and pyrin (sulpyrine and aminopyrine; 0.5 mL/kg; Green Cross Veterinary Products) was done thrice daily for 3 d. The rats were sacrificed 2 wk after tooth extraction, and blood samples were obtained prior to sacrifice. The mandible, including the alveolar bone containing the extraction socket, was obtained and fixed in 10% formalin for subsequent analyses. Micro‐computed tomography (*µ*‐CT) and histological analyses were performed after the surgery to analyze the new bone formation in the extraction socket.

### *µ*‐CT analysis

**FIGURE 1:**
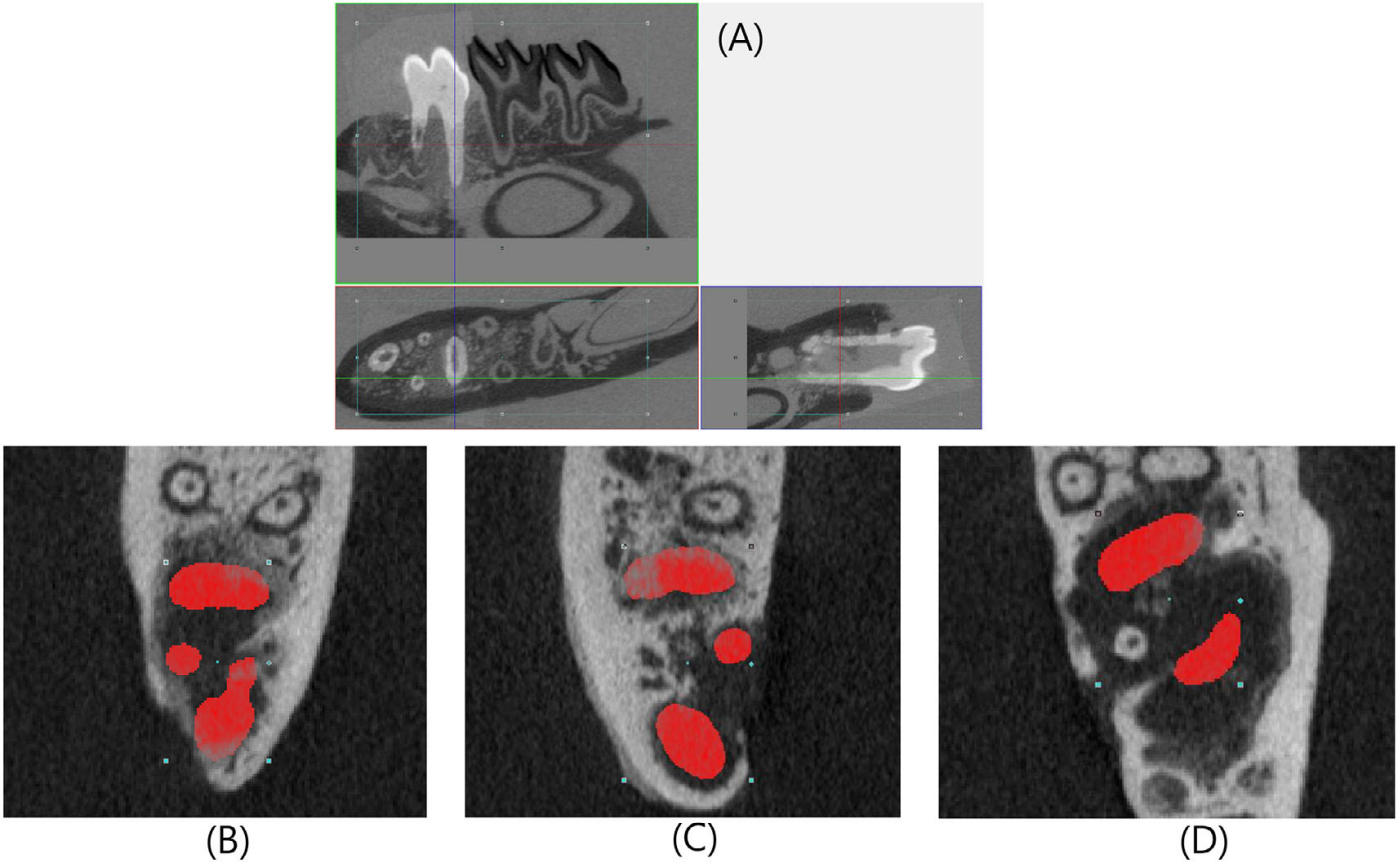
The procedure for setting the region of interest. (A) The image of the tooth image was superimposed on the image of the extraction socket of mandible in 3‐dimensionally. Overlapping image of the (B) 2 M, (C) 10 M, and (D) 18 M groups.

The mandibular specimens, fixed in 10% formalin, were taken using *µ*‐CT at the Center for University‐Wide Research Facilities of Jeonbuk National University. The specimens were taken by a SkyScan 1076 x‐ray scanner (Belgium) with a pixel size of 35 *µ*m. The sets of CT scanner were 100 kV voltage for the x‐ray tube, 100 *µ*A current for the x‐ray source, and 190 ms exposure time. The x‐ray source and detector were rotated by 360° in 0.6°‐increments. The NRecon software (Bruker) was used to perform the 3‐dimensional reconstruction of the specimens. To precisely evaluate the regenerated bone within the extraction socket, a tooth‐based ROI method was employed. First, the extracted tooth from each specimen was scanned under the same conditions. The 3D image of the extracted tooth was then digitally superimposed onto the corresponding image of the mandibular extraction socket (Figure 1). The ROI was defined strictly according to the anatomical outline of the original tooth root space, thereby excluding the surrounding alveolar bone (Figure 1). The ROI was set according to the outline of the original tooth space. BV, bone mineral density (BMD), trabecular thickness (TbTh), and trabecular space (TbSp) of the regenerated bone were evaluated. BMD was calibrated using standard hydroxyapatite phantoms. The grayscale threshold for defining new bone in the extraction socket was set in the range of 70–160.

### Serum biochemical analysis

Blood samples collected from the left jugular vein were subjected to enzyme‐linked immunosorbent assay (ELISA) immediately before surgery at baseline (T0) and 2 wk postoperatively, immediately before sacrifice (T1). The collected blood samples were transported to the laboratory, and the whole blood was centrifuged at 13,000 *g* for 10 min for the separation of the serum. The separated serums were stored at −70°C until serum analysis. Serum samples were analyzed for alkaline phosphatase (ALP, Cat. No. MBS764162; MyBioSource) that total ALP was measured using the specified ELISA kit, CTX‐I (Cat. No: MBS2502522, MyBioSource), OC (Cat. No. MBS2022619, MyBioSource), RANKL (Cat. No: MBS2022170), and TRAP5b (TRAP5b, Cat. No: MBS9901647; MyBioSource) using ELISA kits, in accordance with the manufacturer's instructions.

### Histological examination

The mandibular specimens with alveolar bone were decalcified in 10% EDTA for a period of 4 wk. The samples were then dehydrated using ethanol and xylene. Subsequently, the specimens were sectioned along the sagittal plane through the midline of the extraction socket and embedded in paraffin blocks. The mandible samples were sectioned approximately 4 *µ*m thickness in sagittal plane. The hematoxylin and eosin staining was done and was examined under an Olympus BX51 microscope (Olympus), and digital images were taken using a digital camera (DP‐73; Olympus).

### Immunohistochemistry

Immunohistochemistry (IHC) was performed to evaluate ALP and TRAP expression in the histological sections. The primary antibodies were the anti‐ALP (M190; Takara) and anti‐TRAP (sc‐28,204). The Dako REAL EnVision Detection System (Dako) was used for IHC in accordance with the manufacturer's instructions, and Mayer's hematoxylin (Sigma‐Aldrich) was used for counterstaining. The BX51 microscope (Olympus) and digital camera (DP‐73, Olympus) were used for the examination of the stained slide.

### Statistical analysis

Statistical analyses were performed using SPSS software (Version 25.0, IBM Corp.). For *µ*‐CT analysis, differences among groups were analyzed using one‐way analysis of variance followed by Tukey's post hoc test.

For serum bone turnover markers, generalized estimating equations were employed to analyze longitudinal data. The regression models included age group, time, and age group × time interaction terms, with the 2 M group at baseline (T0) defined as the reference category. Results are expressed as regression coefficients (*β*) with 95% confidence intervals. A *p*‐value <0.05 was considered statistically significant.

## RESULTS

### Analysis of BV, BMD, TbTh, and TbSp

**FIGURE 2:**
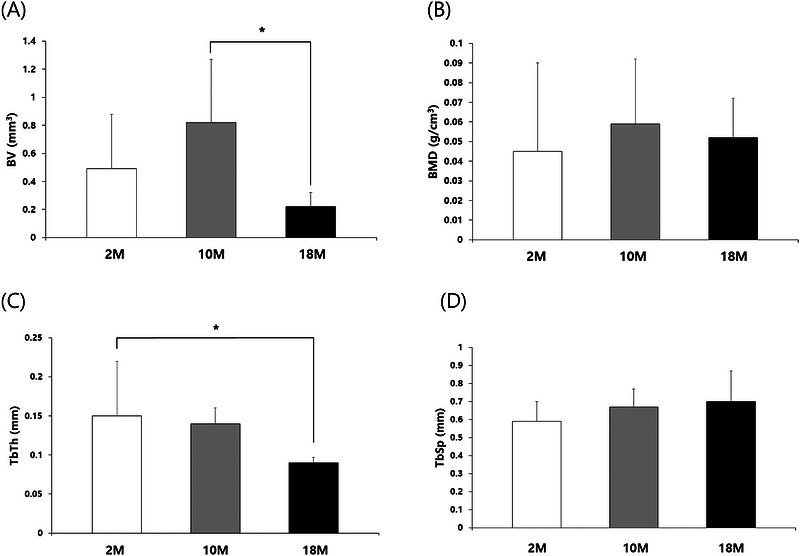
Micro‐computed tomography analysis; (A) bone volume (BV), (B) bone mineral density (BMD), (C) trabecular thickness (TbTh), and (D) trabecular space (TbSp) of 2, 10, and 18 M groups. (*p < 0.05, **p < 0.001).

Figure 2 demonstrates the findings of the *µ*‐CT, including BV, BMD, TbTh, and TbSp. The average BVs of the 2, 10, and 18 M groups were 0.49 ± 0.39, 0.82 ± 0.45, and 0.22 ± 0.10 mm^3^, respectively (Figure 2A). The BV of the 10 M group was significantly greater than that of the 18 M group (*p* = 0.016). The average BMDs of the 2, 10, and 18 M groups were 0.045 ± 0.045, 0.059 ± 0.033, and 0.052 ± 0.020 g/cm^3^, respectively, and there was no significant difference in BMD between the three groups (Figure 2B). The average TbThs of the 2, 10, and 18 M groups were 0.15 ± 0.07, 0.14 ± 0.02, and 0.09 ± 0.007 mm, respectively (Figure 2C). TbTh in the 2 M group was significantly greater than that in the 18 M group (*p* = 0.033). The average TbSps of the 2, 10, and 18 M groups were 0.59 ± 0.11, 0.67 ± 0.10, and 0.70 ± 0.17 mm, and we observed no statistically significant different TbSp between the three groups (Figure 2D).

**FIGURE 3:**
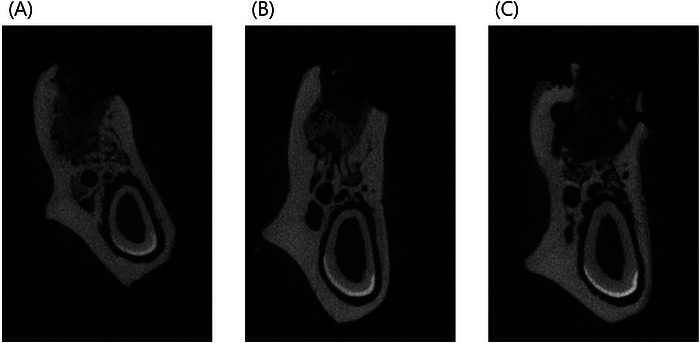
The sectional image of micro‐computed tomography (*µ*‐CT) of extraction socket of mandible. (A) 2 M, (B) 10 M, and (C) 18 M groups. New bone formation is observed in the extraction socket of the 2 and 10 M group. In the 18 M group, bone remodeling is not observed.

Figure 3 depicts sectional *µ*‐CT images of the mandibular specimens crossing the extraction socket. New bone formation was prominent in the extraction sockets of the 2 and 10 M group. In both groups, immature bone filled the socket and was not completely calcified (Figure 3A,B). Unlike the 2 and 10 M groups, the extraction socket of the 18 M group was in an empty space, and new bone formation was not observed. Moreover, a small bone fragment was observed over the alveolar ridge (Figure 3C).

### Expression level of ALP, osteocalcin, RANkL, CTXI, and TRAP5b

The estimates of the effects of age and time on serum bone turnover markers are summarized in Table 1. First, regarding ALP, serum levels remained stable throughout the experiment, with no statistically significant differences observed in baseline levels or temporal changes across all groups (*p* > 0.05). In the case of OC, significant age‐related differences were observed at baseline (T0); both the 10 and 18 M groups exhibited significantly lower levels compared to the 2 M group (all *p* < 0.001). Regarding temporal changes at 2 wk post‐extraction (T1), a significant interaction was found in the 10 M group (*β* = −7.16, *p* = 0.018), indicating a significant decrease compared to the slight increasing trend observed in the 2 M group. For RANKL, although no significant differences were found in baseline levels, the 10 M group exhibited a significant increase at T1 relative to the change observed in the 2 M group (interaction *β* = 0.88, *p* = 0.002). Regarding CTX‐I, baseline levels were significantly lower in the older groups (10 and 18 M) compared to the 2 M group (*p* = 0.010 and *p* = 0.004, respectively). After extraction, the 2 M group showed a decreasing trend in CTX‐I levels (*β* = −1.40), though this did not reach statistical significance (*p* = 0.060). Furthermore, there were no significant differences in the pattern of change among the age groups. Finally, for TRAP5b, the 2 M group showed a significant increase at T1 compared to T0 (*β* = 0.54, *p* = 0.014). Conversely, the 10 M group did not follow this increasing trend, showing a significant reduction in the magnitude of change compared to the 2 M group (interaction *β* = −0.75, *p* = 0.005).

**TABLE 1:** Linear regression estimates of the effects of age and time on serum bone turnover markers.

Outcome	Predictors	Coefficient (*β*)	95% CI (lower, upper)	*p*‐value
ALP (pg/ml)	Intercept (2 M at T0)	137.70	(100.39, 175.01)	<0.001
	Time effect (T1)	−9.933	(−48.85, 28.99)	0.617
	Age group (vs. 2 M at T0)			
	10 M group	19.11	(−25.431, 63.66)	0.400
	18 M group	−5.82	(−46.56, 34.92)	0.779
	Interaction (age × time)			
	10 M × T1	37.61	(−4.70, 79.91)	0.081
	18 M × T1	27.67	(−19.76, 75.10)	0.253
**Osteocalcin (ng/ml)**	Intercept (2 M at T0)	31.66	(28.14, 35.18)	<0.001
	Time effect (T1)	4.19	(−1.00, 9.37)	0.114
	Age group (vs. 2 M at T0)			
	**10 M group**	−**16.82**	(−**21.18,** −**12.45)**	<0.001*
	**18 M group**	−**19.13**	(−**22.99,** −**15.26)**	<0.001*
	Interaction (age × time)			
	**10 M × T1**	−**7.16**	(−**13.12,** −**1.20)**	0.018*
	18 M × T1	6.44	(−1.44, 14.33)	0.109
**RANKL (ng/ml)**	Intercept (2 M at T0)	2.87	(2.50, 3.24)	<0.001
	Time effect (T1)	−0.15	(−0.50, 0.20)	0.394
	Age group (vs. 2 M at T0)			
	10 M group	−0.48	(−1.12, 0.17)	0.146
	18 M group	−0.06	(−0.90, 0.78)	0.885
	Interaction (age × time)			
	**10 M × T1**	**0.88**	**(0.33, 1.43)**	0.002*
	18 M × T1	0.3	(−0.73, 1.32)	0.573
**CTX‐I (ng/ml)**	Intercept (2 M at T0)	7.12	(5.96, 8.28)	<0.001
	Time effect (T1)	−1.4	(−2.86, 0.06)	0.060
	Age group (vs. 2 M at T0)			
	**10 M group**	−**1.68**	(−**2.94,** −**0.41)**	0.010*
	**18 M group**	−**2.38**	(−**4.02,** −**0.74)**	0.004*
	Interaction (age × time)			
	10 M × T1	0.11	(−1.48, 1.70)	0.893
	18 M × T1	0.9	(−0.84, 2.63)	0.309
**TRAP5b (U/l)**	Intercept (2 M at T0)	1.47	(1.21, 1.72)	<0.001
	**Time effect (T1)**	**0.54**	**(0.11, 0.96)**	0.014*
	Age group (vs. 2 M at T0)			
	10 M group	0.13	(−0.29, 0.54)	0.557
	18 M group	−0.02	(−0.37, 0.33)	0.897
	Interaction (age × time)			
	**10 M × T1**	−**0.75**	(−**1.27,** −**0.23)**	0.005*
	18 M × T1	−0.33	(−0.77, 0.12)	0.154

*Note*: Data were estimated using linear regression models with generalized estimating equations (GEE). The 2 M group at baseline (T0) was used as the reference. Values are presented as regression coefficients (*β*) and 95% confidence intervals (CI). *p*‐values < 0.05 are considered statistically significant and are indicated in bold with an asterisk (*).

Abbreviations: 10 M, 10‐month‐old group; 18 M, 18‐month‐old group; 2 M, 2‐month‐old group; ALP, alkaline phosphatase; CI, confidence interval; CTX‐I, C‐terminal telopeptide of type I collagen; RANKL, receptor activator of nuclear factor kappa‐B ligand; TRAP5b, tartrate‐resistant acid phosphatase 5b.

### Histological examination of the new bone

**FIGURE 4:**
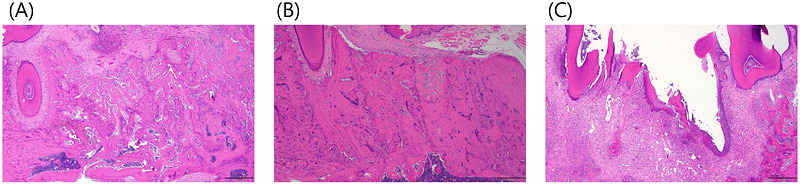
Hematoxylin and eosin staining image of (A) 2 M, (B) 10 M, and (C) 18 M groups. In the 2 M group, immature new bone regeneration is observed in the tooth extraction socket covered with the gingival tissue. In the 10 M group, the extraction socket is almost occupied with new bone (original magnification 40×, scale bar = 500 *µ*m).

Figure 4 depicts the histological sagittal sectional images of each group. In the 2 M group, new immature bone regeneration was observed in the tooth extraction socket space, which was covered with gingival tissue without inflammation (Figure 4A). In the 10 M group, the tooth extraction socket was almost completely occupied with new bone and the gingival tissue healed. The most favorable bone regeneration was observed in the 10 M group (Figure 4B). In the 18 M group, unlike the preceding groups, the extraction socket space was empty and new bone regeneration was difficult to find. A large number of inflammatory cells were present along the extraction socket, which was in an open state without gingival tissue growth (Figure 4C).

### ALP and TRAP5b levels via IHC analysis

**FIGURE 5:**
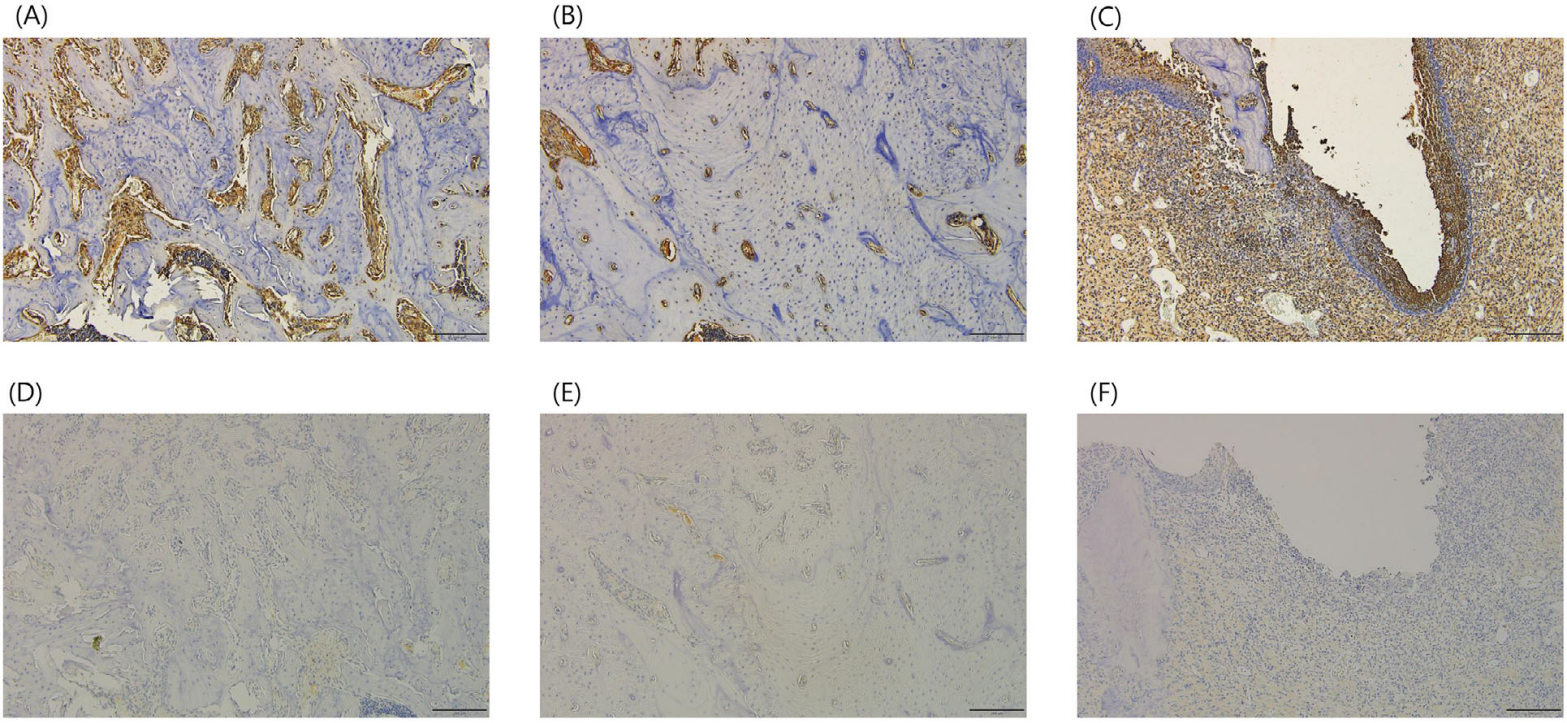
Immunohistochemical staining of alkaline phosphatase (ALP) (A–C) and TRAP (D–F) in each group. (A and D) 2 M, (B and E) 10 M, and (C and F) 18 M group. ALP expression is detected in the osteogenic cells within the newly formed bone matrix of the extraction socket in the 2 and 10 M groups, with greater expression in the 2 M group. No distinct TRAP expression is present in any group. (magnification 100×, scale bar = 200 *µ*m).

Figure 5 depicts the expression of ALP and TRAP5b in new bone. ALP is expressed in the newly formed bone matrix around osteogenic cells. ALP expression was observed in newly formed bone in the extraction socket in the 2 and 10 M group and was the highest in the 2 M group, followed by the 10 M group and 18 M group. Moreover, in the 18 M group, osteoblastic cells were sparsely distributed (Figure 5A–C). TRAP5b expression was not prominent in the bones of any of the groups (Figure 5E,F).

## DISCUSSION

In this study, we compared the alveolar bone healing and regeneration capacities among rats of different ages to investigate the effects of aging on extraction socket healing. Previous studies have investigated the effects of aging on bone healing in the maxillofacial region, including the maxillary palate, mandibular bone defect, and condyle [5, 15, 19]. However, the effects of aging on alveolar bone healing and regeneration after tooth extraction remain unclear. The alveolar bone, a part of the mandible, anchors periodontal fibers to the bone and supports mechanical forces during mastication [20]. Preserving alveolar bone dimensions after tooth extraction is crucial for successful dental restoration procedures [21]. Adequate BV is necessary for prosthetic treatment and dental implant placement. Given the aging population, understanding the alveolar bone remodeling process after tooth extraction in older adults is vital to ensure effective dental restoration. In this study, we aimed to evaluate the effects of aging on specific patterns of bone regeneration in the mandibular extraction socket.

Bone remodeling in the extraction socket was assessed using *µ*‐CT 2 wk after the tooth extraction. To evaluate the bone occupying the space previously filled by the tooth roots, a ROI was defined for analysis of bone parameters. Previously, the ROI of the tooth extraction site included not only the extraction socket space but also the surrounding alveolar bone [22]. In this study, we aimed to calculate the tooth space without including the surrounding bone. To define the tooth‐extracted space as the ROI, we first scanned the extracted tooth in the same manner as for the mandibular samples. The extracted tooth image was then superimposed onto the extraction socket of the mandible to create an overlapping image in 3‐dimensionally (Figure 1). This enabled us to define an ROI that included only the tooth‐extracted space surrounding alveolar bone. Our analysis method has not been previously reported in studies that have utilized *µ*‐CT analysis. Although this approach requires significant time, technical effort, and additional software handling, it offers a more accurate evaluation of changes in the extraction socket and facilitates comparisons of CT parameters among groups.

On the basis of *µ*‐CT findings, extraction socket regeneration was evaluated using BV, BMD, TbTh, and TbSp. The BV of the 10 M group was significantly greater than that of the 18 M group (*p* = 0.016); similarly, the TbTh of the 2 M group was significantly higher than that of the 18 M group (*p* = 0.033). In addition, the average BV and TbTh of the 18 M group were the lowest among the three groups (Figure 2). Bone regeneration in the extraction socket was more prominent in the younger group than that in the older group. This tendency was observed in the sectional *µ*‐CT image, where regenerated bone was observed in the extraction socket along the socket wall (Figure 3). In some samples from the 18 M groups, a destructive bone along the socket wall was observed. When a biomaterial is implanted in a cranial defect in an aged rat model, aging impairs bone repair ability and decreases osteogenic potential [23]. Similarly, in a rat mandibular condyle fracture model, bone healing was significantly delayed in older rats [5]. The extraction socket healing differs from craniofacial bone healing due to the involvement of stem cells originating from residual PDL fibroblasts [7]. These PDL cells contribute uniquely to bone regeneration in the socket through their osteogenic potential [9]. However, despite these unique biological characteristics, the effects of aging on the extraction socket followed a similar trend to cranial and mandibular regeneration. In our study, *µ*‐CT analysis clearly demonstrated that bone regeneration was more prominent in the younger (2 and 10 M) groups compared to the older group (Figure 3), suggesting that aging negatively affects bone regeneration even in the extraction socket.

Interestingly, our previous study using a mandibular bone defect model reported contrasting results, where aged rats exhibited greater bone regeneration compared to younger rats. In that study, the favorable healing in older animals was attributed to the anatomical advantage of a thicker mandibular bone, which provided a more voluminous bony wall [15]. However, in the present study, we utilized a “tooth ROI” technique to strictly define the defect space based on the extracted tooth morphology. By controlling these local anatomical variations via the tooth ROI method, the systemic negative effects of aging—characterized by reduced metabolic activity—became the dominant factor, resulting in the observed decline in bone healing in the older group.

The analysis of serum bone turnover markers revealed that younger animals generally exhibited higher bone formation activity and a more distinct metabolic response to tooth extraction compared to older animals (Table 1). Serum levels of the bone formation marker OC were significantly elevated in the younger group compared to the older group. However, serum ALP levels remained stable across all groups. Serum levels of ALP and OC vary according to age and human pubertal stage [24]. Generally, serum ALP and OC levels are elevated in the 20–29 yr old group to the older group and tend to decrease with increasing age [25, 26]. Our animal study results are similar to those of clinical studies. Serum levels of bone formation markers were elevated in the 2 and 10 M groups compared to the 18 M group, which corresponded with the *µ*‐CT findings: greater BV and TbTh in both the 2 and 10 M groups as well as greater TbSp in the 18 M group.

Regarding bone resorption markers, RANKL, a member of the TNF superfamily, controls osteoclast differentiation by binding to RANK on osteoclast precursors [27]. RANKL expression is stimulated by various cytokines produced by osteoblasts. Increased RANKL levels activate osteoclast differentiation and promote bone resorption [28]. In this study, although a transient increase in RANKL levels was observed in the 10 M group at T1 (post‐extraction), baseline levels were similar among all groups, and this marker did not show a consistent pattern correlating with the delayed healing observed in the older groups. In contrast, serum CTX‐I levels were significantly higher in the 2 M group compared to the older groups at baseline, reflecting the naturally high bone turnover rate characteristic of younger individuals [1, 4]. Regarding TRAP5b, the 2 M group exhibited a significant increase following tooth extraction; however, the 10 M group failed to show this elevation, suggesting a blunted osteoclastic response to surgical trauma with aging. This finding is consistent with previous reports indicating that aging significantly delays bone healing and remodeling in the mandible [5].

Bone regeneration in the extraction socket was observed histologically in the 2 and 10 M groups (Figure 4A,B). The osseous contents filled the socket and were covered with gingival tissue. In the 2 M group, osteogenic tissue and trabecular bone were formed in the socket, and fibrous tissue and blood vessels were present on the new bone (Figure 4A). Bone healing and regeneration in the extraction socket were more prominent in the 2 and 10 M groups than in the 18 M group. Histologically, alveolitis was observed in the 18 M group, similar to that observed in *µ*‐CT images. However, the effect of aging on extraction socket healing remains poorly understood. However, the occurrence of post‐extraction complications significantly depended on patient age. Dry sockets are more prevalent in older patients [29, 30]. ALP expression was observed in osteogenic cells in the lacunae of mineralized new bone material in the 2 and 10 M groups (Figure 5).

Bone regeneration and healing capacity decline with age due to age‐related changes in the bone [1]. This aging effect also influences craniomaxillofacial bone metabolism. However, studies that have investigated the effect of aging on the healing of the tooth extraction socket remain limited. Bone formation in the extraction socket is essential for installing dental implants [31]. Bone loss in the extraction socket after tooth extraction is inevitable and can be affected by systemic diseases and functional factors of the stomatognathic system [7]. Aging significantly alters bone metabolism and compromises alveolar bone healing in the maxillofacial region, which is a critical determinant for the successful rehabilitation of the dentition [4, 20].

In this study, we investigated the effects of aging on bone regeneration and healing in the extraction socket of alveolar bone. This study aimed to apply these results in the clinical field of dentistry. We found that the younger (2 and 10 M) group demonstrated increased bone formation in the extraction socket of the alveolar bone compared to the older (18 M) group, with increased levels of bone turnover markers. In conclusion, aging affects bone regeneration and healing in extraction sockets. Younger rats exhibited greater new bone formation in the extraction socket of alveolar bone. Therefore, aging should be considered during the dental rehabilitation of individuals with tooth loss. Although this is the first study to report on the effect of aging on alveolar bone healing, there are some limitations, including the small sample size used in the animal experiment. The sample size was insufficient to generalize the results of our study. Furthermore, there may be variations in health conditions in each animal due to old age. Further studies using a larger number of animals are required in the future.

## AUTHOR CONTRIBUTIONS

**Conceptualization**: Dae Ho Leem and Hyun Seok. **Methodology**: Kyung Su Shin, Han Wool Kwak, Jong‐Seong Chae, Si‐On Choi, Suah Han, and Hwa Young Yu. **Validation**: Kyung Su Shin, Jong‐Seong Chae, and Si‐On Choi. **Formal analysis**: Kyung Su Shin, Jong‐Seong Chae, and Si‐On Choi. **Investigation**: Kyung Su Shin, Suah Han, and Hwa Young Yu. **Writing—original draft**: Han Wool Kwak. **Writing—review and editing**: Kyung Su Shin, Dae Ho Leem, and Hyun Seok. **Visualization**: Jong‐Seong Chae, Si‐On Choi, and Han Wool Kwak. **Project administration**: Han Wool Kwak.

## CONFLICT OF INTEREST STATEMENT

The authors declare no conflicts of interest.

## Data Availability

The sharing of the data of the micro‐CT and serum analysis is available on request. The data on our study can be shared by reasonable request to the corresponding author.
